# Periostin‐related progression of different types of experimental pulmonary hypertension: A role for M2 macrophage and FGF‐2 signalling

**DOI:** 10.1111/resp.14249

**Published:** 2022-03-22

**Authors:** Takashi Yoshida, Tetsutaro Nagaoka, Yuichi Nagata, Yoshifumi Suzuki, Takeo Tsutsumi, Sachiko Kuriyama, Junko Watanabe, Shinsaku Togo, Fumiyuki Takahashi, Masakazu Matsushita, Yusuke Joki, Hakuoh Konishi, Satoshi Nunomura, Kenji Izuhara, Simon J. Conway, Kazuhisa Takahashi

**Affiliations:** ^1^ Department of Respiratory Medicine Juntendo University Faculty of Medicine and Graduate School of Medicine Tokyo Japan; ^2^ Department of Internal Medicine and Rheumatology Juntendo University Faculty of Medicine and Graduate School of Medicine Tokyo Japan; ^3^ Department of Cardiovascular Medicine Juntendo University Faculty of Medicine and Graduate School of Medicine Tokyo Japan; ^4^ Division of Medical Biochemistry, Department of Biomolecular Sciences Saga Medical School Saga Japan; ^5^ Wells Center for Pediatric Research Indiana University School of Medicine Indianapolis Indiana USA

**Keywords:** experimental pulmonary hypertension, fibroblast growth factor, M2 macrophage, periostin, vascular remodelling

## Abstract

**Background and objective:**

Remodelling of pulmonary arteries (PA) contributes to the progression of pulmonary hypertension (PH). Periostin, a matricellular protein, has been reported to be involved in the development of PH. We examined the role of periostin in the pathogenesis of PH using different types of experimental PH.

**Methods:**

PH was induced by vascular endothelial growth factor receptor antagonist (Sugen5416) plus hypoxic exposure (SuHx) and venous injection of monocrotaline‐pyrrole (MCT‐P) in wild‐type (WT) and periostin^−/−^ mice. Pulmonary haemodynamics, PA remodelling, expression of chemokines and fibroblast growth factor (FGF)‐2, accumulation of macrophages to small PA and the right ventricle (RV) were examined in PH‐induced WT and periostin^−/−^ mice. Additionally, the role of periostin in the migration of macrophages, human PA smooth muscle (HPASMCs) and endothelial cells (HPMVECs) was investigated.

**Results:**

In PH induced by SuHx and MCT‐P, PH and accumulation of M2 macrophage to small PA were attenuated in periostin^−/−^ mice. PA remodelling post‐SuHx treatment was also mild in periostin^−/−^ mice compared to WT mice. Expression of macrophage‐associated chemokines and FGF‐2 in lung tissue, and accumulation of CD68‐positive cells in the RV were less in SuHx periostin^−/−^ than in SuHx WT mice. Periostin secretion in HPASMCs and HPMVECs was enhanced by transforming growth factor‐β. Periostin also augmented macrophage, HPASMCs and HPMVECs migration. Separately, serum periostin levels were significantly elevated in patients with PH compared to healthy controls.

**Conclusion:**

Periostin is involved in the development of different types of experimental PH, and may also contribute to the pathogenesis of human PH.

## Graphical abstract

We demonstrated that periostin was involved in the development of different types of experimental pulmonary hypertension (PH). M2 macrophage and fibroblast growth factor‐2 signalling appeared to contribute to the periostin‐related progression of PH. In addition, the levels of serum periostin were significantly higher in patients with PH.

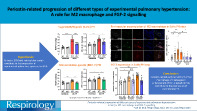

## INTRODUCTION

The pathogenesis of pulmonary arterial hypertension (PAH) involves abnormal vasoconstriction and vascular remodelling in pulmonary arteries (PA), and the remodelling of PA plays an important role in the development of advanced PAH. Several vasodilators have been developed for PAH^1^; however, there are no drugs targeting vascular remodelling; investigation of the mechanisms responsible for vascular remodelling in PAH is essential for drug development.

Periostin, an extracellular matrix protein, is involved in the fibrosis of diseased tissues and plays a role in the pathogenesis of various diseases.^2^ It has been reported that periostin regulates macrophage, vascular endothelial cell and smooth muscle cell functions.^3^, ^4^ Although several groups have demonstrated the contribution of periostin to the progression of pulmonary hypertension (PH),^4^, ^5^, ^6^ its exact role is still not well understood. Therefore, we examined the role of periostin in the progression of PH using different types of experimental PH models.

## METHODS

### Experimental PH models

The PH mouse model was established using Sugen 5416 (vascular endothelial growth factor receptor‐1,‐2 inhibitor) and chronic hypoxic exposure (10% O_2_) for up to 3 weeks (SuHx), and intravenous monocrotaline‐pyrrole (MCT‐P) as previously described^7^, ^8^ (Figure S1A in the Supporting information).

### Haemodynamic measurements

Right heart catheterization of mice was performed as described in Appendix S1 in the Supporting Information.

### Histology and immunohistochemistry of the lung and RV

Formalin‐fixed paraffin‐embedded inflated left lungs and right ventricles (RV) were used for staining. Lung tissue sections were stained with Elastica van Gieson stain (EVG). Immunohistochemical staining of the lung and RV was performed as described in Appendix S1 in the Supporting Information.

### Assessment of periostin and macrophage accumulation in peri‐small PA

Right lungs were stored for mRNA analysis, while left lungs were inflated with Optimal Cutting Temperature Compound (Sakura Finetek Japan, Tokyo, Japan) to be processed for immunohistochemistry for frozen sections as described in Appendix S1 in the Supporting Information.^9^

### Quantitative real‐time PCR

We examined wild‐type (WT) normoxia and SuHx (3 days, 7 days, 3 weeks), periostin^−/−^ normoxia and SuHx (3 days, 7 days, 3 weeks) mice. Quantitative real‐time PCR was performed as described in Appendix  S1 and Table S2 in the Supporting Information.

### Cell culture

Primary human pulmonary microvascular endothelial cells (HPMVECs) and primary human pulmonary arterial smooth muscle cells (HPASMCs) were obtained from Lonza (Basel, Switzerland). U937 and RAW 264.7, human and mouse cell lines, respectively, were obtained from KAC Co., Ltd (Kyoto, Japan).

### Reagents

Information regarding reagents used in the present study is provided in Appendix S1 in the Supporting Information.

### ELISA

Plasma periostin levels and the concentrations of periostin in culture supernatants were measured using a sandwich ELISA and a custom‐made anti‐periostin monoclonal antibody (clone numbers SS19A and SS19C for plasma, clone numbers SS18A and SS17B for medium) as described in the Supporting Information.^10^

### Chemotaxis assay

HPASMCs, HPMVECs, U937 pre‐treated with PMA^10^ and RAW264.7 cell chemotaxis were assessed using the 48‐well micro chemotaxis chambers (Neuroprobe, Inc., Gaithersburg, MD, USA), as previously described.^11^

### Serum periostin levels in patients with PH

Serum periostin levels were assessed using blood samples from PH patients and healthy volunteers. PH was defined as mean pulmonary arterial pressure (MPAP) greater than 20 mm Hg on right heart catheterization. Detailed patient information is presented in Table S1 in the Supporting Information.

### Statistical analysis

Data are presented as means ± SD. Statistical analyses were performed using one‐way ANOVA (Tukey's multiple comparisons test, Dunnett's multiple comparisons test and Dunn's multiple comparisons test), two‐way ANOVA (Sidak's multiple comparisons test), log‐rank (Mantel–Cox) test and *t*‐test (Prism 8; GraphPad Software, La Jolla, CA, USA). Differences were considered significant at *p* <0.05.

## RESULTS

### Periostin contributes to the development of PH in different mouse models

**FIGURE 1:**
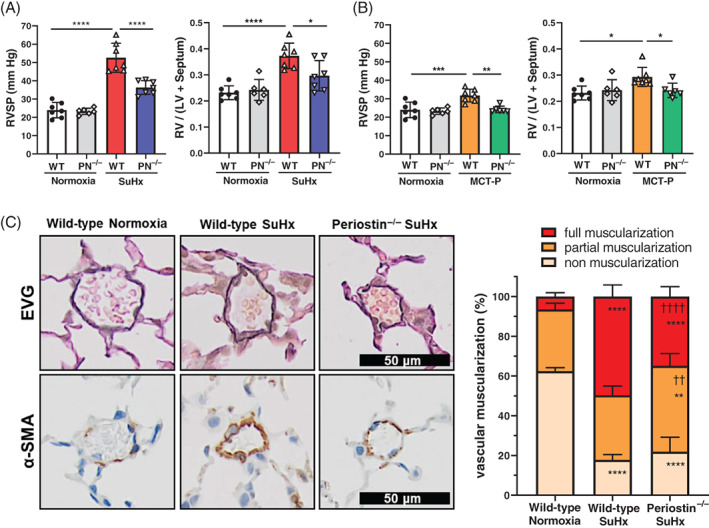
Pulmonary haemodynamics and muscularization of small pulmonary arteries in pulmonary arterial hypertension mice, induced with Sugen5416 and chronic hypoxia (SuHx) and monocrotaline pyrrole (MCT‐P). Effect of periostin gene knockout on RVSP, RV hypertrophy (RV/[LV + S]) in SuHx wild‐type (WT) and periostin^−/−^ (PN^−/−^) mice (A), and in MCT‐P WT mice and MCT‐P periostin^−/−^ mice (B); RVSP, right ventricular systolic pressure; RV/(LV + S), right ventricle/(left ventricle + septum) weight ratio. Data are presented as means ± SD (*n* = 6 or 7 per group). Statistical comparisons were conducted using one‐way ANOVA followed by Tukey's multiple comparisons test versus WT normoxia or WT SuHx and are represented above each plot. *p <0.05, ***p <0.001.****p <0.0001. (C) Representative photomicrographs of Elastica van Gieson staining and quantitative analysis of the pulmonary small vessels muscularization in WT normoxia, WT SuHx and periostin^−/−^ SuHx groups; 50–100 pulmonary vessels (with an outer diameter of less than 50 μm) per section were analysed, blinded to the tissue source. Scale bar = 50 μm. Data are presented as means ± SD (*n* = 6 per group) and statistical comparisons were performed using two‐way ANOVA followed by Sidak's multiple comparisons test. **p <0.01, ****p <0.0001 versus respective WT normoxia groups; ^††^ *p* <0.01, ^††††^ *p* <0.0001 versus respective WT SuHx groups

RV systolic pressure (RVSP) and RV/left ventricle (LV) + septum weight ratio (RV/LV + S) in SuHx and MCT‐P WT mice were significantly increased compared to normoxic mice; however, RVSP and RV/LV + S in periostin^−/−^ PH‐induced mice were significantly lower than that in SuHx and MCT‐P WT mice (Figure 1A,B).

Next, we evaluated the muscularization of the small PA using the EVG and α‐smooth muscle actin (SMA) staining of small PA in normoxic WT, SuHx WT and SuHx periostin^−/−^ mice (Figure 1C). The number of small PA with full muscularization was significantly higher in SuHx WT mice compared to normoxic WT mice, and the muscularization of small PA with PH induction was significantly decreased in SuHx periostin^−/−^ mice. Expression of periostin in the whole lung homogenate and remodelled small PA were increased in SuHx WT mice (Figure S1B,C in the Supporting Information).

### Expression of macrophage‐related chemokine and phenotype markers in lung tissues of SuHx mice

**FIGURE 2:**
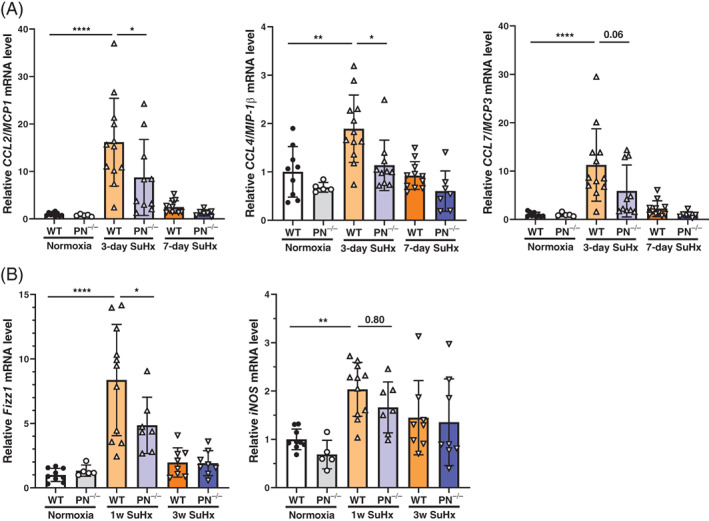
mRNA expression of macrophage markers and chemokines associated with macrophage migration to pulmonary arteries in SuHx wild‐type (WT) and periostin^−/−^ mice. (A) mRNA levels of CCL2, CCL4 and CCL7 in whole lung tissue of WT and periostin^−/−^ normoxia mice, SuHx WT and periostin^−/−^ mice. RNA was extracted from lung tissue 3 or 7 days after SuHx treatment and applied to quantitative real‐time (RT) PCR. Data are presented as fold changes relative to the 18S ribosomal mRNA level; PN^−/−^, periostin^−/−^. Results are presented as means ± SD from five to 12 samples. Statistical comparisons were conducted using multiple groups ANOVA followed by Tukey's multiple comparisons test. *p <0.05, **p <0.01, ****p <0.0001. (B) The mRNA expression levels of macrophage markers in lung tissues from WT and periostin^−/−^ SuHx mice. RNA was extracted from lung tissue 1 or 3 weeks after SuHx treatment and quantitative RT‐PCR was performed to assess the mRNA levels of inducible nitric oxide synthase (iNOS) (M1 macrophage) and Fizz1 (M2 macrophage) marker genes. Data are presented as fold changes relative to the 18S ribosomal mRNA levels. Results are presented as means ± SD from five to 11 samples. Statistical comparisons of multiple groups were conducted using ANOVA followed by Tukey's multiple comparisons test. *p <0.05, **p <0.01, ****p <0.0001

The expression levels of CCL2 (MCP1) and CCL4 (MIP‐1β), chemokines involved in macrophage migration in whole lung tissue, were significantly higher in WT than in periostin^−/−^ mice 3 days after SuHx treatment, and the expression of CCL7 (MCP3) had a similar pattern. Chemokine expression gradually decreased by day 7 after SuHx treatment (Figure 2A).

Using whole lung tissue, we evaluated the mRNA levels of inducible nitric oxide synthase (iNOS) and found in inflammatory zone‐1 (Fizz1), markers of M1 and M2 macrophages, respectively. While the expression of iNOS was higher in SuHx WT mice than in normoxic WT mice 1‐week post‐SuHx treatment, the levels of iNOS were similar in SuHx WT and periostin^−/−^ mice. At the same time, Fizz1 levels were significantly higher in SuHx WT mice than in normoxic WT mice at 1 week time point, while Fizz1 levels were significantly lower in SuHx periostin^−/−^ compared to SuHx WT mice (Figure 2B).

### M1 and M2 macrophage accumulation in peri‐small PA of SuHx and MCT‐P‐treated mice

**FIGURE 3:**
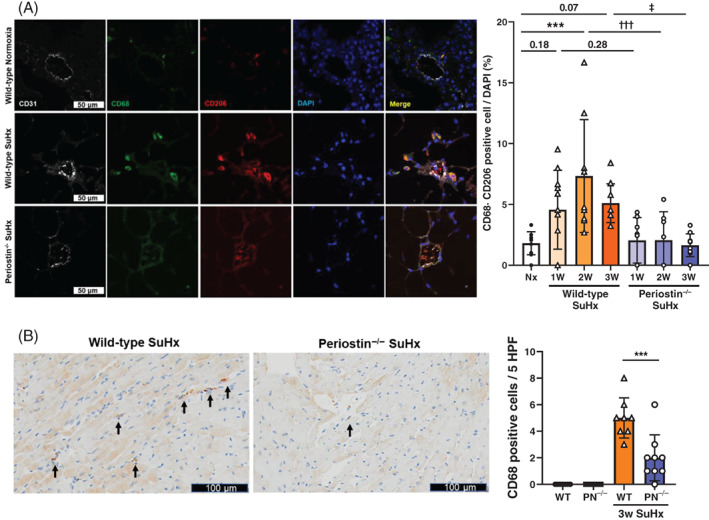
Localization of CD68‐ and CD206‐positive macrophages to pulmonary arteries (PAs) and CD68‐positive cells to right ventricle (RV) in SuHx wild‐type (WT) and periostin^−/−^ mice. (A) Representative fluorescence immunostaining of peri‐PAs from WT normoxia (Nx), WT SuHx and periostin^−/−^ SuHx mice 2 weeks post‐stimulation with Sugen5416 and hypoxic exposure. Lung sections were stained with anti‐CD31 Ab, anti‐CD68 Ab and anti‐CD206 Ab (M2 macrophage marker), and counterstained with DAPI. Representative immunofluorescence images are shown (white, CD31; green, CD68; red, CD206; blue, DAPI, yellow, co‐localization of CD68 with CD206); scale bar 50 μm. The percentage of CD68+ and CD206+ double‐positive cells was calculated as the ratio of yellow cells to blue (DAPI) cells. Results are presented as means ± SD from 10 to 12 vessels per group. Statistical comparisons were conducted using ANOVA followed by Tukey's multiple comparisons test. ***p <0.001; ^†††^ *p* <0.001; ^‡^ *p* <0.05. (B) Representative sections of RV tissue from WT SuHx and periostin^−/−^ SuHx mice analysed by CD68 immunostaining. Sections of RV tissue were stained with anti‐CD68 Ab. The number of CD68‐positive cells (pointed by black arrows) per five high‐power fields (5 HPF) was calculated. Results are presented as means ± SD from six to nine samples. Scale bar = 100 μm. Statistical comparisons of multiple groups were conducted using ANOVA followed by Tukey's multiple comparisons test. ***p <0.001

To investigate the accumulation and phenotypes of macrophages around small PA, lung tissues were stained for CD68, a macrophage and white blood cell marker, and CD206, an M2 macrophage marker. The numbers of CD68‐ and CD206‐positive macrophages in the peri‐small PA were significantly increased in SuHx WT mice compared to normoxic WT mice at 2 weeks after SuHx treatment, the time point before PH would even be established. Increased M2 macrophage was reduced in SuHx periostin^−/−^ mice at the same time point (Figure 3A). The accumulation of M2 macrophages was similarly observed in MCT‐P WT mice and significantly decreased in MCT‐P periostin^−/−^ mice (Figure S2B in the Supporting Information). Cells positive for CD11c, an M1 macrophage marker, did not accumulate in small PA in both WT and periostin^−/−^ mice with PH‐induced SuHx and MCT‐P (Figure S2A,C in the Supporting Information).

### Macrophage accumulation in RV of SuHx mice

Expression of CD68‐positive cells in the RV of SuHx WT mice was significantly higher than in normoxic WT mice, and its expression was also significantly decreased in SuHx periostin^−/−^ mice (Figure 3B).

### Periostin expression and its effect on HPMVEC, HPASMC and macrophage migration

Periostin concentrations in culture supernatants were corrected for cell number. In HPASMCs, periostin concentration was significantly higher after stimulation with transforming growth factor (TGF)‐β2, but not in response to PDGF‐BB or TNF‐α (Figure S3A in the Supporting Information). In HPMVECs, periostin concentration was also significantly increased in response to TGF‐β2, but not to IL‐1β or TNF‐α (Figure S3B in the Supporting Information).

**FIGURE 4:**
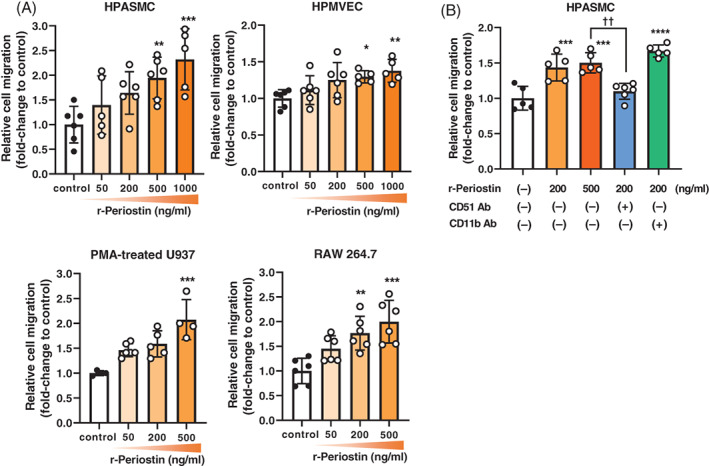
Regulation of migration by periostin in human pulmonary arterial smooth muscle cells (HPASMCs), human pulmonary microvascular endothelial cells (HPMVECs) and human and mice macrophages. (A) Dose‐dependent effects of periostin on chemotaxis in HPASMCs, HPMVECs, phorbol 12‐myristate 13‐acetate (PMA)‐treated U937 and RAW264.7 cells. (B) The number of migrated HPASMCs following treatment with various concentrations of periostin in the presence or absence of anti‐CD11b and anti‐CD51 antibodies. The concentration of recombinant periostin (r‐periostin) varied from 50 to 1000 ng/ml. The chemotaxis vertical axis shows the number of migrated cells per five high‐power fields (5 HPF) as the fold change compared to control (media alone). Results are presented as means ± SD from four to six samples. Statistical comparisons were conducted using multiple groups ANOVA followed by Dunnett's multiple comparisons test, Dunn's multiple comparisons test or Tukey's multiple comparisons test. *p <0.05, **p <0.01, ***p <0.001, ****p <0.0001; ^††^ *p* <0.01

Chemotaxis assays showed that periostin increased the migration of HPASMCs and HPMVECs in a concentration‐dependent manner. Next, we examined cell migration in macrophages. First, we induced the differentiation of U937 into macrophages using phorbol 12‐myristate 13‐acetate (PMA) as previously described.^11^ The migration of PMA‐induced U937 and RAW264.7 macrophages was also enhanced by periostin in a dose‐dependent manner (Figure 4A). To identify cell surface receptors that bind to periostin, we used anti‐CD51 (anti‐αV receptor) and anti‐CD11b (anti‐αM receptor) antibodies to block cell migration. The migration of HPASMCs was significantly attenuated by anti‐CD51 antibody, but not by anti‐CD11b antibody (Figure 4B).

### Expression of inflammatory cytokines and FGF‐2 in SuHx mice

**FIGURE 5:**
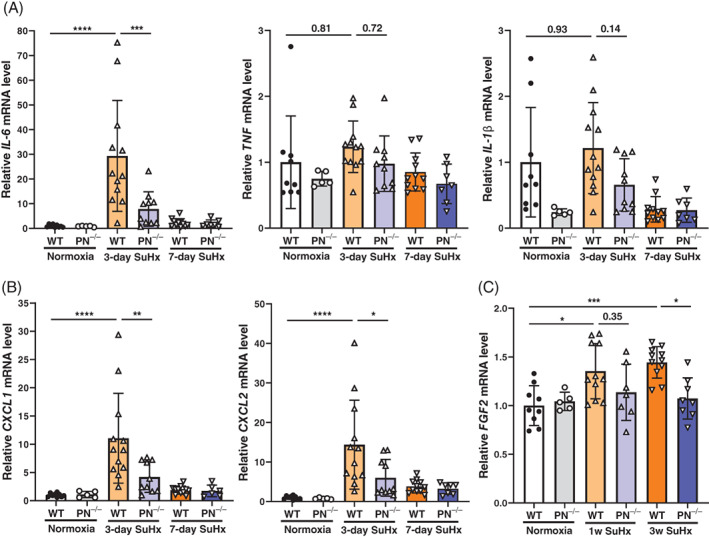
Expression of inflammatory cytokines and neutrophil‐associated chemokines in whole lung tissue of SuHx wild‐type (WT) and periostin^−/−^ mice. mRNA levels of (A) IL‐6, TNF‐α, IL‐1β and (B) CXCL1, CXCL2 and (C) Fibroblast growth factor‐2 in whole lung tissue from SuHx WT and periostin^−/−^ (PN^−/−^) mice. RNA was extracted from lung tissue 3 or 7 days or 3 weeks after SuHx treatment and quantitative real‐time PCR was performed. Data are presented as fold changes relative to the 18S ribosomal mRNA level; PN^−/−^, periostin^−/−^. Results are presented as means ± SD from five to 12 samples. Statistical comparisons of multiple groups were conducted using ANOVA followed by Tukey's multiple comparisons test. *p <0.05, **p <0.01, ***p <0.001, ****p <0.0001

We assessed the mRNA levels of inflammatory cytokines involved in the pathogenesis of PAH (Figure 5A), as well as chemokines involved in neutrophil migration (CXCL1, CXCL2) (Figure 5B), in whole lung tissues. The expression levels of IL‐6, CXCL1 and CXCL2 were significantly higher in SuHx WT mice at day 3 time point than in normoxic WT mice; however, SuHx periostin^−/−^ mice had significantly lower expression levels of these inflammatory markers.

We also examined the mRNA of fibroblast growth factor (FGF)‐2 in lung tissue of SuHx mice. Expression of FGF‐2 was higher in SuHx WT mice than in normoxic WT mice at 3 days and 3 weeks after the treatment with SuHx. Augmented expression of FGF‐2 in WT mice at 3 weeks after SuHx treatment was significantly attenuated in periostin^−/−^ mice (Figure 5C).

### Serum periostin levels in patients with or without PH

**FIGURE 6:**
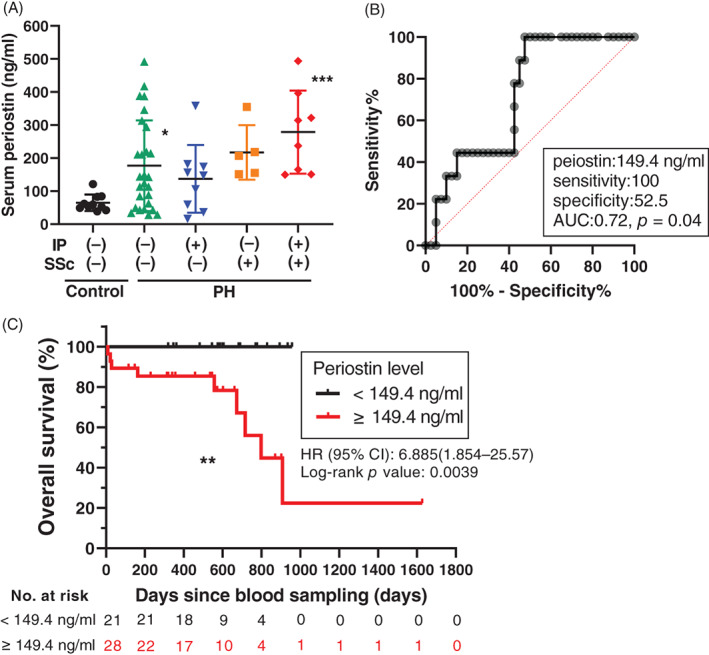
Serum periostin levels in patients with pulmonary hypertension (PH). (A) Serum periostin levels according to the presence of interstitial pneumonia (IP) and systemic sclerosis (SSc). IP(+), presence of interstitial lung disease; IP(−), absence of interstitial lung disease; SSc(+), presence of SSc; SSc(−), absence of SSc. Results are presented as means ± SD. Statistical comparisons of multiple groups were performed using ANOVA followed by Tukey's multiple comparisons test. *p <0.05, **p <0.01, ***p <0.001 versus control. (B) Receiver operating characteristic curve for best‐fit value of serum periostin level to predict mortality. (C) Overall survival from the time of blood sampling in relation to periostin serum levels in 49 patients with PH. Patients with periostin levels measuring 149.4 ng/ml or higher had a poor prognosis

Serum periostin concentrations in patients with PH (MPAP > 20 mm Hg, *n* = 49, see Table S1 in the Supporting Information) were significantly increased compared to healthy controls (*n* = 10). Higher serum levels of periostin in patients with interstitial pneumonitis^12^, ^13^ and systemic scleroderma^14^ have been demonstrated. Therefore, we excluded patients with these diseases from analysis. Nevertheless, serum periostin concentrations were significantly higher in patients with PH than in healthy controls (Figure 6A). Receiver operating characteristic curve analysis identified a serum periostin cut‐off threshold of 149.4 ng/ml as the best fit for a significant predictive performance (as assessed by area under the curve of 0.71; *p* = 0.04; Figure 6B) that maximized specificity and sensitivity for mortality. Using this cut‐off threshold to dichotomize the periostin variable, the overall survival of the periostin‐high group was significantly worse than that of the periostin‐low group (Figure 6C).

## DISCUSSION

In the present study, we demonstrated the contribution of periostin to the development of PH and M2 macrophage recruitment in different types of experimental PH induced by SuHx and MCT‐P. In addition, we also showed that periostin may regulate the expression of FGF‐2 signalling during the progression of PH induced by SuHx.

Periostin promotes fibrosis in diseased tissues, and also regulates cellular functions by binding to integrin αvβ3 and αvβ5 receptors.^15^, ^16^ Periostin contributes to the development of various respiratory diseases,^17^ including interstitial pneumonitis^12^, ^18^ and bronchial asthma.^19^, ^20^, ^21^, ^22^, ^23^ Several reports also suggest that periostin contributes to the pathogenesis of PH. Periostin was expressed in the remodelled PA of rat^24^ and human PH.^5^ Several inflammatory cytokine and growth factors including TGF‐β, TNF‐α, IL‐1β, IL‐6, leukotriene B4 and pletelet‐derived growth factor (PDGF) were shown to be important indicators for the progression of PA remodelling in PAH.^25^, ^26^, ^27^ In the present study, TGF‐β mostly contributes to the production of periostin from HPMVECs and HPASMCs, the finding consistent with previous reports.^6^, ^28^ Recently, Nie et al. showed that periostin regulated the function of PA endothelial cells and revealed an important role of an hypoxic inducible factor (HIF)‐1α‐dependent pathway in the periostin‐related progression of PH.^4^ Here, using a chemotaxis assay, we demonstrated that periostin induced the migration of HPMVECs and HPASMCs via integrin αv receptors. Previous reports showed the contribution of FGF‐2 in the development of PH.^29^, ^30^, ^31^ We also demonstrated that increased expression of FGF‐2 in lung tissue of SuHx WT mice was significantly reduced in periostin^−/−^ WT mice at 3 weeks after the treatment with SuHx. This result suggests that periostin may be involved in the progression of PH through increased FGF‐2 signalling that follows early macrophage accumulation.

Lower chemokine production and the accumulation of macrophages due to the deletion of periostin has been reported in pulmonary fibrosis^18^ and atherosclerotic plaques.^3^ It is well known that peri‐PA inflammatory cells play an important role in the progression of vascular remodelling in PAH.^25^, ^32^, ^33^, ^34^ A recent report showed that deletion of macrophages by intrabronchial clodronate acid attenuated the PH progression and PA remodelling in SuHx mice, indicating that macrophages play an important role in the development of PH.^35^ We demonstrated that the expression of chemokines associated with macrophage migration was elevated 3 days post‐SuHx treatment in WT mice, and this increase was significantly inhibited in periostin^−/−^ mice. The accumulation of macrophages in the peri‐PA was the highest at 2 weeks after SuHx and MCT‐P treatment, and the majority of these cells were M2 macrophages. Previous reports showed that smooth muscle cell proliferation was augmented via M2 macrophage‐induced CCL2 signalling,^33^ whereas the mRNA levels of iNOS, an M1 macrophage marker, were not different between WT and periostin^−/−^ mice after SuHx treatment. These results suggest that the contribution of M2 macrophages could be greater than that of M1 macrophages in periostin‐induced PA remodelling in PH. Furthermore, involvement of periostin in cardiac fibrosis has been demonstrated in monocrotaline‐induced PH rats.^36^, ^37^, ^38^ The present study showed that increased accumulation of CD68‐positive cells, including macrophage, in RV of SuHx WT mice was significantly reduced in SuHx periostin^−/−^ mice. This result indicates that periostin may also contribute to the RV fibrosis via macrophage recruitment.

Our results also suggest that periostin may be involved in neutrophil‐mediated inflammation, as elevated CXCL1 and CXCL2 mRNA levels in SuHx WT mice were attenuated in SuHx periostin^−/−^ mice. Furthermore, the lung tissue expression of IL‐6 was already at its peak on day 3 post‐SuHx treatment, suggesting that periostin was affecting other cells, rather than macrophages. These results indicated that periostin was involved in the regulation of several inflammatory pathways, in addition to macrophages.

We observed that serum periostin levels in PAH/PH patients, excluding interstitial pneumonitis and systemic scleroderma patients, were still higher than in healthy controls. In addition, patients with serum periostin levels higher than 149.4 ng/ml at diagnosis had a significantly poorer prognosis. On the other hand, the clinical assessment of the present study was retrospectively performed and the sample size was also insufficient. Additionally, the control group is not properly matched to the PH patient group. Thus, the present clinical data should be considered as just pilot data.

There were several limitations in our present study. First, the role of M2 macrophages in the enhancement of FGF‐2 signalling is unclear. Second, confirmation of protein expression of cytokines and chemokines was insufficient. Third, the mechanism of progression of myocardial fibrosis induced by periostin is still uncertain. Fourth, clinical data in this study will require further validation and generalizability to confirm the efficacy of periostin as a biomarker for PH prognosis.

In conclusion, we revealed that lack of periostin attenuated the development of different types of PH induced by SuHx and MCT‐P. Early recruitment of M2 macrophage and subsequent enhancement of FGF‐2 signalling may contribute to the progression of these experimental PH models.

## CONFLICT OF INTEREST

None declared.

## AUTHOR CONTRIBUTION

**Takashi Yoshida:** Conceptualization (equal); data curation (lead); formal analysis (lead); investigation (lead); methodology (lead); project administration (lead); resources (lead); software (lead); visualization (lead); writing – original draft (equal); writing – review and editing (supporting). **Tetsutaro Nagaoka:** Conceptualization (lead); formal analysis (supporting); funding acquisition (lead); methodology (equal); project administration (equal); supervision (lead); validation (lead); visualization (supporting); writing – original draft (supporting); writing – review and editing (lead). **Yuichi Nagata:** Formal analysis (supporting); software (supporting). **Yoshifumi Suzuki:** Data curation (supporting); formal analysis (supporting); software (supporting). **Takeo Tsutsumi:** Formal analysis (supporting); software (supporting). **Sachiko Kuriyama:** Conceptualization (supporting); formal analysis (supporting); software (supporting); validation (equal). **Junko Watanabe:** Investigation (supporting); methodology (supporting). **Shinsaku Togo:** Investigation (supporting); methodology (supporting). **Fumiyuki Takahashi:** Funding acquisition (supporting); writing – review and editing (supporting). **Masakazu Matsushita:** Resources (equal); writing – review and editing (supporting). **Yusuke Joki:** Resources (equal); writing – review and editing (supporting). **Hakuoh Konishi:** Resources (equal); writing – review and editing (supporting). **Satoshi Nunomura:** Investigation (supporting); methodology (supporting). **Kenji Izuhara:** Investigation (supporting); methodology (supporting). **Simon J. Conway:** Funding acquisition (supporting); resources (equal). **Kazuhisa Takahashi:** Funding acquisition (supporting); writing – review and editing (supporting).

## HUMAN AND ANIMAL ETHICS APPROVAL DECLARATION

Patient samples were collected and analysed in accordance with the Declaration of Helsinki and the study was conducted with the approval of the institutional review board of Juntendo University Hospital (approved number 18‐033, Tokyo, Japan). We obtained informed consent for the collection of clinical samples.

All animal experimental and surgical procedures were approved by the Institutional Committee for the Use and Care of Laboratory Animals in Juntendo University (approval no: 310013, Tokyo, Japan), and were performed in accordance with the U.S. National Institutes of Health Guide for the Care and Use of Laboratory Animals.

## Supporting information


**Supporting information**.

**Visual Abstract**. Periostin‐related progression of different types of experimental pulmonary hypertension: A role for M2 macrophage and FGF‐2 signalling

## Data Availability

The data that support the findings of this study are available on request from the corresponding author. The data are not publicly available due to privacy or ethical restrictions.
