# Supplementary material for: Periostin‐related progression of different types of experimental pulmonary hypertension: A role for M2 macrophage and FGF‐2 signalling
Source: Respirology. 2022 Mar 22;27(7):529–38. doi: 10.1111/resp.14249 (PMC9313806; doi:10.1111/resp.14249)
Supplement: Supplementary file 1 — Supporting information. [file RESP-27-529-s001.docx]

**SUPPORTING INFORMATION**

Periostin-related progression of different types of

experimental pulmonary hypertension:

A role for M2 macrophage and FGF-2 signaling

Takashi Yoshida^1^, Tetsutaro Nagaoka^1^, Yuichi Nagata^1^, Yoshifumi Suzuki^1^,

Takeo Tsutsumi^1^, Sachiko Kuriyama^1^, Junko Watanabe^1^, Shinsaku Togo^1^,

Fumiyuki Takahashi^1^, Masakazu Matsushita^2^, Yusuke Joki^3^, Hakuoh Konishi^3^,

Satoshi Nunomura^4^, Kenji Izuhara^4^, Simon J Conway^5^, Kazuhisa Takahashi^1^

^1^Department of Respiratory Medicine, Juntendo University Faculty of Medicine and Graduate School of Medicine, Tokyo, Japan

^2^Department of Internal Medicine and Rheumatology, Juntendo University Faculty of Medicine and Graduate School of Medicine, Tokyo, Japan

^3^Department of Cardiovascular Medicine, Juntendo University Faculty of Medicine and Graduate School of Medicine, Tokyo, Japan

^4^Division of Medical Biochemistry, Department of Biomolecular Sciences, Saga Medical School, Saga, Japan

^5^Wells Center for Pediatric Research, Indiana University School of Medicine, Indianapolis, Indiana, USA

**Appendix S1: SUPPLEMENTARY METHODS**

**Preparation of SuHx PH mouse**

Male periostin^-/-^ mice (C57BL/6 background) were generated as previously described.^1^ Male C57BL/6 mice (8-9 weeks old, 22.5±2.5 g, Sankyo Labo Service Corporation, INC., Tokyo, Japan) and periostin^-/-^ mice (22.5±2.5 g) were injected once weekly with Sugen 5416 (20 mg/kg; R&D Systems, Minneapolis, MN, USA) subcutaneously and exposed to hypobaric hypoxia (370 mmHg, 10% O_2_) for 3 days, 1 week, 2 weeks, or 3 weeks (3 day SuHx, 1 w SuHx, 2 w SuHx, 3 w SuHx, respectively). After exposure to hypoxia and weekly SU5416 injections, mice were used for further experiments at the indicated time points (Figure S1 (A)).

**Preparation of monocrotaline-pyrrole induced PH mice**

monocrotaline-pyrrole (MCT-P) (5mg/kg; Santa Cruz, Dallas, TX, USA) was intravenously administered via the tail vein. The evaluations of the pathophysiology of PH were performed at 14 days after the MCT-P injection. After hemodynamic evaluations, the animals were euthanized, and the heart and lungs were extracted for further evaluations (Figure S1A). MCT-P was dissolved in dimethylformamide and the stock solution of MCT-P was kept at -80°C until use.

**Hemodynamic measurements**

Wild type (WT) normoxia mice (n=7), periostin^-/-^ normoxia mice (n=6), WT 3 w SuHx mice (n=7), periostin^-/-^ 3 w SuHx mice (n=7), WT MCT-P mice (n=7) and periostin^-/-^ MCT-P mice (n=6) were used for hemodynamic measurements. Each mouse was anesthetized using continuous inhaled isoflurane (2.0%–2.5%). A 1.4 Fr catheter (Millar, Houston, TX, USA) was inserted into the right ventricle (RV) via the right jugular vein to measure RV systolic pressure (RVSP) using the PowerLab data acquisition system (AD Instruments, Dunedin, New Zealand). Heart rates (HR) were continuously monitored, and mice with HR less than 300 beats/minute were excluded. After the hemodynamic measurements, all mice were euthanized by intraperitoneal administration of pentobarbital sodium (200mg/kg), and hearts and lungs were collected for RV/left ventricle (LV) + septum weight ratio (RV/LV+S) measurements to analyze RV hypertrophy, and for histological evaluations. Right lungs were stored for protein measurements, and left lungs were inflated with 10% buffered formalin at a constant pressure of 20 cm H_2_O for histological analyses.

**Histology and immunohistochemistry of lung and right ventricle**

The BenchMark GX (Ventana Medical Systems, Inc. Oro Valley, AZ, USA) was used. Antibodies used for immunohistochemistry were as follows: α-Smooth muscle actin (α-SMA) (1:500, Abcam, Cambridge, UK), and Goat Anti-Rabbit Immunoglobulins/Biotin (1:200, Agilent Technologies, Santa Clara, CA, USA). Slides were examined using VS120 Virtual Slide Microscope and image viewer software OlyVIA (Olympus Life Science Solutions, Tokyo, Japan). Between 50 and 100 small PAs (10–50 μm diameter) per whole left lobe were counted and assessed for the degree of circumferential α-SMA-positive staining indicative of muscularization. Vessels were scored as follows: no evidence of muscularization (α-SMA-positive area < 25%), partial muscularization (25-75%), and full muscularization (> 75%). All samples were blinded and scored at the same time.

We used formalin-fixed paraffin embedded RV tissues from WT normoxia mice (n=7), periostin^-/-^ normoxia mice (n=6), WT 3 w SuHx mice (n=8), periostin^-/-^ 3 w SuHx mice (n=9). The BenchMark GX was used. Antibodies used for immunohistochemistry were as follows: CD68 (1:250, Abcam), and Horse Anti-Rabbit IgG Polymer Kit (Vector Laboratories, Inc, Burlingame, CA, USA). Macrophage Infiltration was assessed by counting the number of CD68 positive cells in five high-power fields.

CD68 have been the most frequently used immunohistochemical marker to identify macrophages, while it is also found on monocytes, neutrophils, basophils, and large lymphocytes. Thus, we considered cells to be macrophages that merged with expression of M1 or M2 markers in addition to CD68 positive.

**Western blotting analysis**

Lung tissues were lysed in the radio-immunoprecipitation assay (RIPA) buffer containing protease and phosphatase inhibitors, and the lysates were subjected to western blotting, as previously described.^2^ Transferred membranes were incubated with the custom-made anti-periostin monoclonal antibody (1 µg/ml, clone no. SS19C)^3^ or anti-β-actin antibody (1:1000, Cell Signaling Technology, Danvers, MA, USA). Western blot images were acquired using a fluorescence imager and quantified using the Image Lab software (Bio-Rad, Hercules, CA, USA). The densitometric signal for each protein was normalized to that of β-actin.

**Assessment of periostin and macrophage infiltration in SuHx PH mouse model**

Anti-periostin monoclonal antibody (clone no. SS19C)^3^ and polyclonal antibodies against CD68 (BioLegend, San Diego, CA, USA), von Willebrand factor (vWF) (Abcam), CD31 (BD, Franklin Lakes, NJ, USA), smooth muscle actin (SMA) (Sigma-Aldrich, St. Louis, MO, USA), and CD206 (Proteintech, Rosemont, IL, USA) were used. For secondary antibodies, Alexa Fluor 488-labeled anti-mouse antibodies, Alexa Fluor 555-labeled anti-mouse antibodies, Alexa Fluor 555-labeled anti-rabbit antibodies, Alexa Fluor 555-labeled anti-sheep antibodies, and Alexa Fluor 647-labeled anti-rat antibodies (Invitrogen, Carlsbad, CA, USA) were used. For nuclear staining, 4’,6-diamidino-2-phenylindole (DAPI, Invitrogen) was used. For quantitative analysis, data sets were rendered and analyzed using ImageJ, Imaris (Bitplane, Belfast, UK), and Zen software (Carl Zeiss, Oberkochen, Germany).

**Quantitative real-time PCR**

RNA was extracted from the lungs using the RNeasy Plus Mini Kit (Qiagen, Hilden, Germany). Extracted RNA was converted to cDNA using the High-Capacity cDNA Reverse Transcription Kit with RNase Inhibitors (Thermo Fisher Scientific, Waltham, MA, USA) with 500–1000 ng starting material per reaction. qPCR was performed on the StepOnePlus™ Real-Time PCR System using PowerUp™ SYBR™ Green Master Mix (Thermo Fisher Scientific). qPCR data were analyzed using the 2^–∆∆Ct^ method;^4^ 18S ribosomal RNA was used as a control. specific dye. The primers which use in present study are listed in table S2.

**Reagents**

Recombinant human platelet-derived growth factor-BB (PDGF-BB) was purchased from Sigma-Aldrich. Recombinant human tumor necrosis factor-α (TNF-α), transforming growth factor β2 (TGF-β2), and interleukin (IL)-1β were purchased from Peprotech (Rocky Hill, CT, USA). Phorbol 12-myristate 13-acetate (PMA) was purchased from Abcam. Anti-human CD11b antibody and anti-human CD51 antibody were purchased from BioLegend.

**ELISA**

To investigate whether growth factors upregulate the secretion of periostin, HPASMCs at 70–80% confluency were cultured in 6-well plates, serum-starved for 6 h, and then incubated in Smooth Muscle Cell Basal Medium (SMBM, Lonza) supplemented with 0.1% FCS + PDGF-BB (30 ng/ml), 0.1% FCS + TNF-α (5 ng/ml), or 0.1% FCS + TGF-β2 (2.5 ng/ml). Next, cells were incubated for 0 h, 24 h, and 48 h and periostin concentrations were measured. In a similar experiment, HPMVECs at 70–80% confluency were cultured in Microvascular Endothelial Cell Growth Medium-2 (EGM, Lonza) supplemented with IL-1β (4 ng/ml), TNF-α (2 ng/ml), or TGF-β2 (2.5 ng/ml) and periostin concentrations were measured using a sandwich ELISA and a custom-made anti-periostin monoclonal antibody (clone no. SS18A and SS17B) as previously described.^3^

**Chemotaxis assay**

Recombinant human or mouse periostin (R&D Systems) was placed in the bottom chamber (48-well micro chemotaxis chambers, Neuroprobe, Inc., Gaithersburg, MD, USA) as the chemoattractant. The top and bottom wells were separated by an 8 μM pore filter (Nucleopore, Pleasanton, CA, USA). The chambers with HPASMCs were incubated at 37°C in a humidified atmosphere containing 5% CO_2_ for 6 h, HPMVECs for 12 h, PMA-treated U937, and RAW264.7 for 24 h. Next, cells on the top of the filter were removed by scraping. The cells that had migrated through the filter were then fixed, stained with DiffQuick (Sysmex, Kobe, Tokyo), and mounted on glass microscope slides. Migration was assessed by counting the number of cells in five high-power fields. The following media were used as negative controls: SMBM + 5% FCS for HPASMCs, EGM for HPMVECs, and RPMI1640 + 10% FCS for PMA-treated U937 and RAW264.7 cells. The results from each well were normalized to that of negative controls. Moreover, to assess whether chemotaxis is mediated by integrin receptors, anti-CD11b and anti-CD51 antibodies, anti-integrin αM receptor and anti-integrin αV receptor, respectively, were added into the upper chamber of HPASMCs at a concentration of 10 μg/ml, and the chemotaxis assay was performed as mentioned above.

**Periostin serum levels in patients with pulmonary hypertension**

The levels of serum periostin, as a potential diagnostic and prognostic biomarker, were assessed using blood samples from PH patients and healthy volunteers. Serum samples were collected from 49 PH patients (25–84 y.o), attending the Departments of Respiratory Medicine, Cardiovascular Medicine, and Rheumatology at the Juntendo University Hospital between 2012 and 2020 as part of the routine diagnostic workup, and were included in the biomaterial bank after obtaining a written informed consent. Blood samples were taken from healthy volunteers (n=10, 34–62 y.o) as controls after obtaining a written informed consent.

**References for Supporting Information**

1. Rios H, Koushik SV, Wang H, Wang J, Zhou HM, Lindsley A et al. Periostin null mice exhibit dwarfism, incisor enamel defects, and an early-onset periodontal disease-like phenotype. *Mol Cell Biol*. 2005;25:11131-44.

2. Tsutsumi T, Nagaoka T, Yoshida T, Wang L, Kuriyama S, Suzuki Y et al. Nintedanib ameliorates experimental pulmonary arterial hypertension via inhibition of endothelial mesenchymal transition and smooth muscle cell proliferation. *PLoS One*. 2019;14:e0214697.

3. Ohta S, Okamoto M, Fujimoto K, Sakamoto N, Takahashi K, Yamamoto H et al. The usefulness of monomeric periostin as a biomarker for idiopathic pulmonary fibrosis. *PLoS One*. 2017;12:e0174547.

4. Livak KJ, Schmittgen TD. Analysis of relative gene expression data using real-time quantitative PCR and the 2(-Delta Delta C(T)) Method. *Methods*. 2001;25:402-8.

**Figure S1. Periostin expression was upregulated in the lung tissues of SuHx mice**


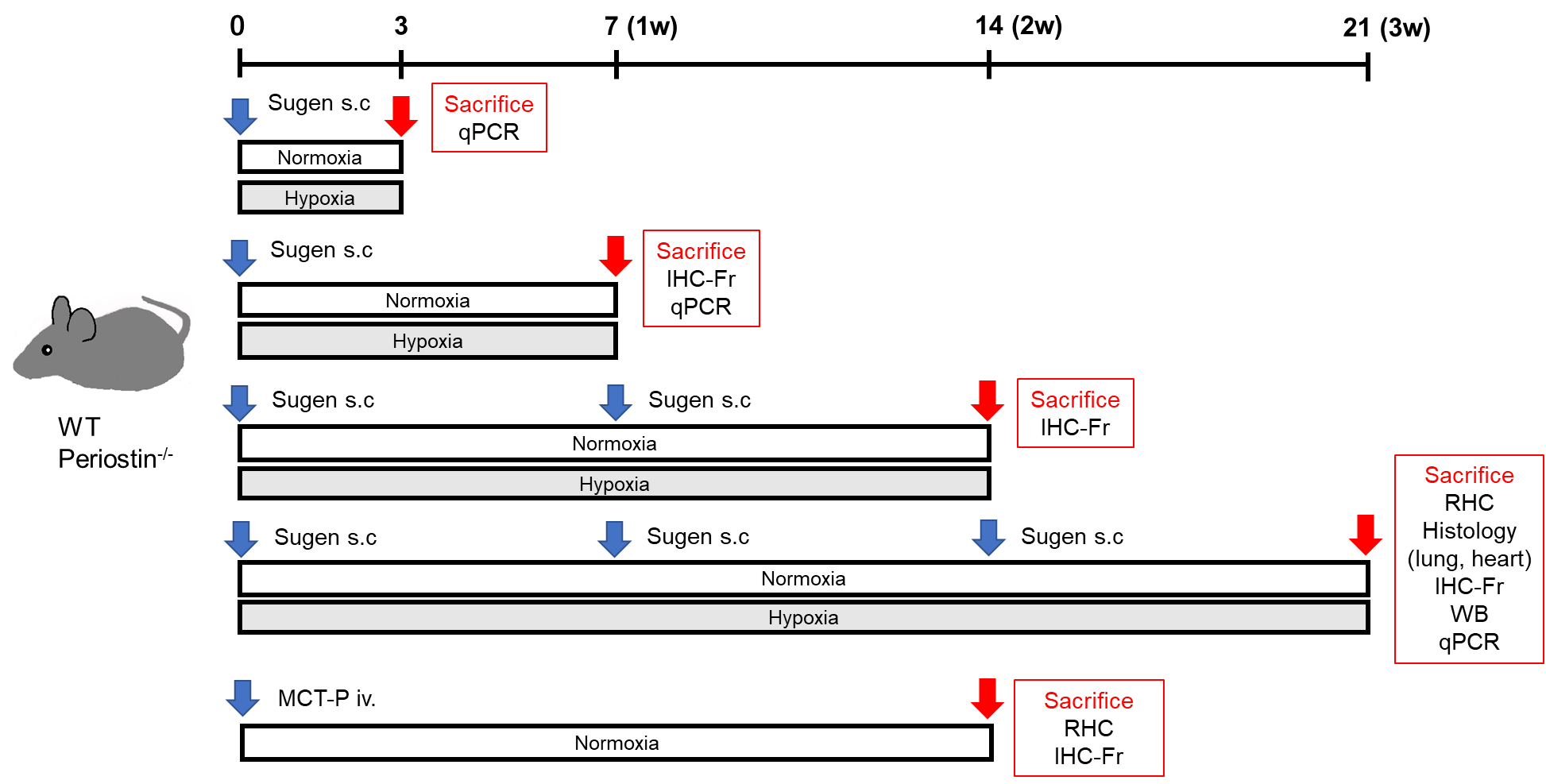


**A**


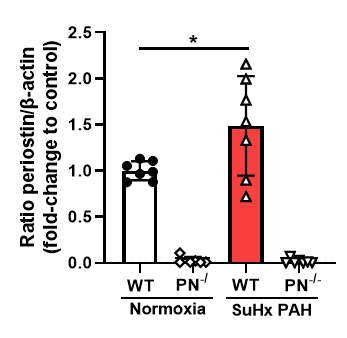


**B**


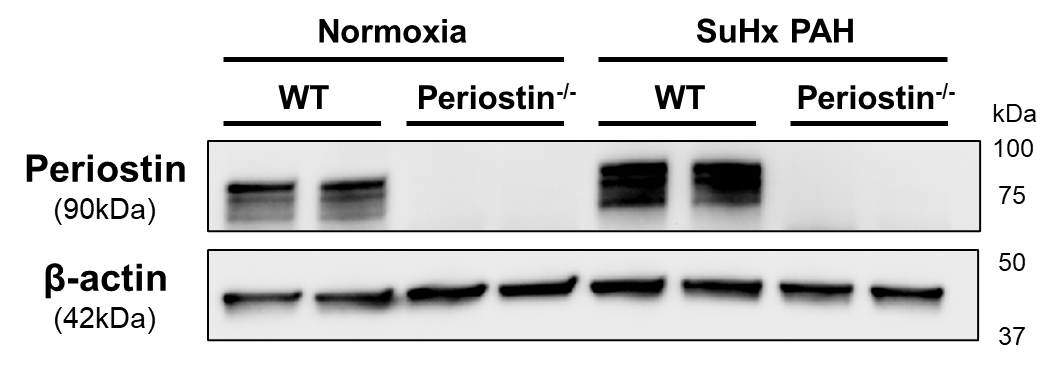


**C**


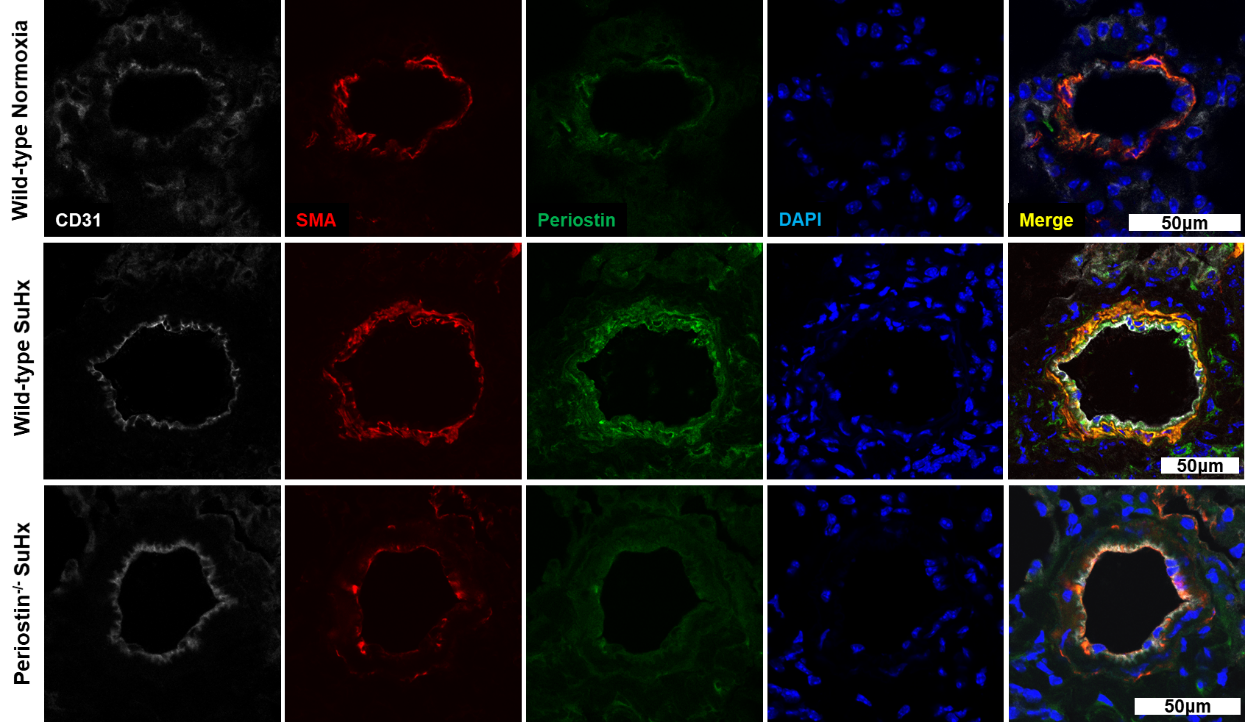


(A) Schematic representation of mouse experiments (timelines and conditions).

(B) Representative western blot and quantification of periostin protein levels in lung tissues from mice (n=6, 7 per group). β-actin was used as loading control. The protein expression of periostin in whole lung tissues was significantly greater in SuHx WT mice than in normoxic control WT mice. No expression of periostin was detected in either periostin^-/-^ normoxia mice or periostin^-/-^ SuHx mice. Results are presented as means ± SD. Statistical comparisons were performed using multiple groups ANOVA followed by Tukey’s multiple comparisons test. *P<0.05.

(C) Representative fluorescence immunostaining of periostin, CD31, α-SMA, and DAPI in the lungs of WT normoxia, WT SuHx, and periostin^-/-^ SuHx mice. Lung sections were stained with anti-periostin Ab, anti-CD31 Ab, and anti-α-SMA Ab (green, periostin; white, CD31; red, α-SMA; blue, DAPI). Periostin was expressed in medial and intimal lesions of WT normoxic control mice, while its expression was greater in WT SuHx mice.

**Figure S2. Accumulation of macrophages to small PA in WT and periostin^-/-^ mice induced PH by SuHx and MCT-P**


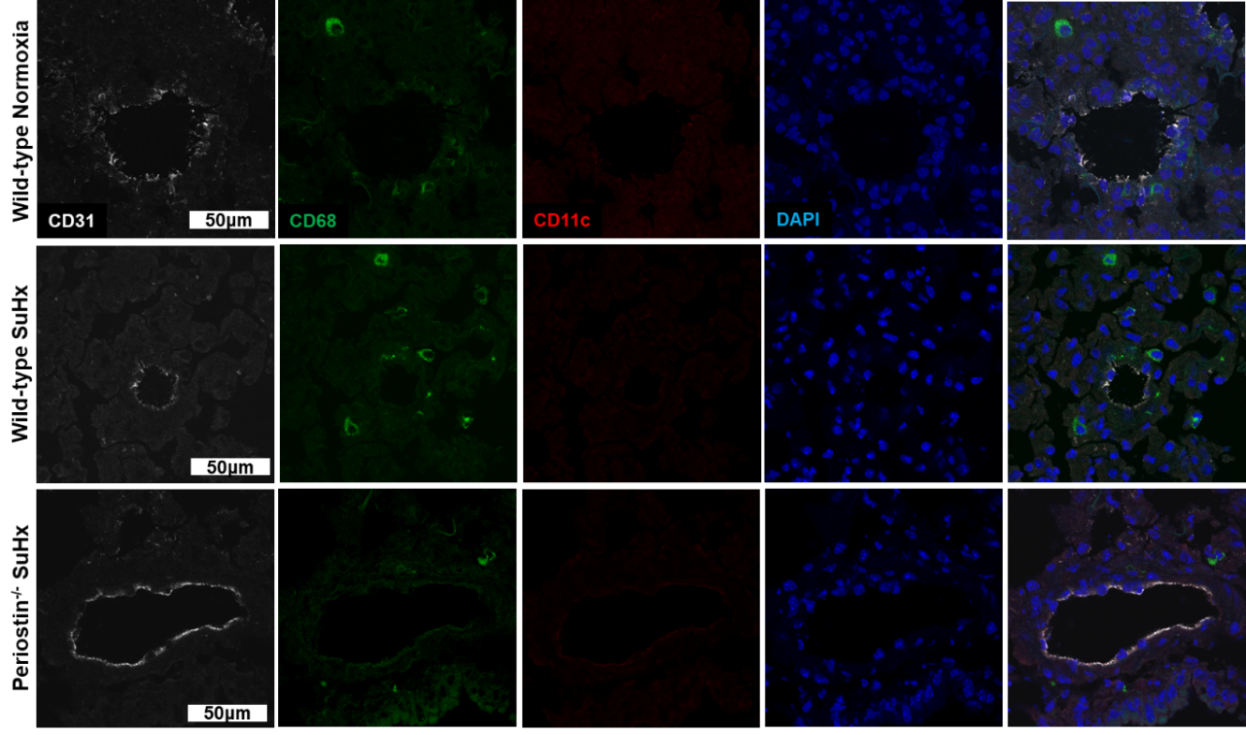


**A**

**B**


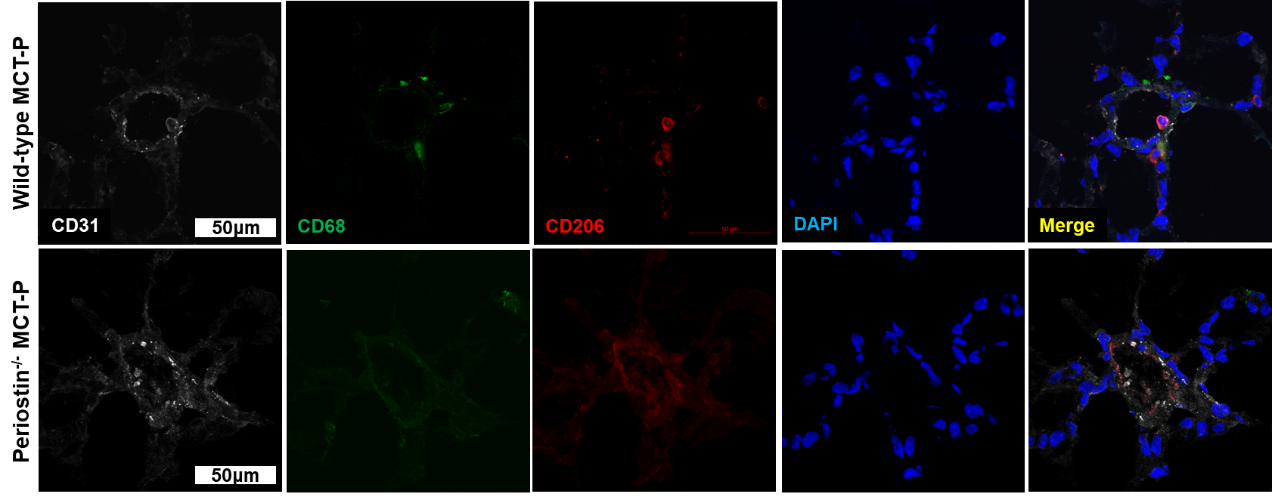


**C**


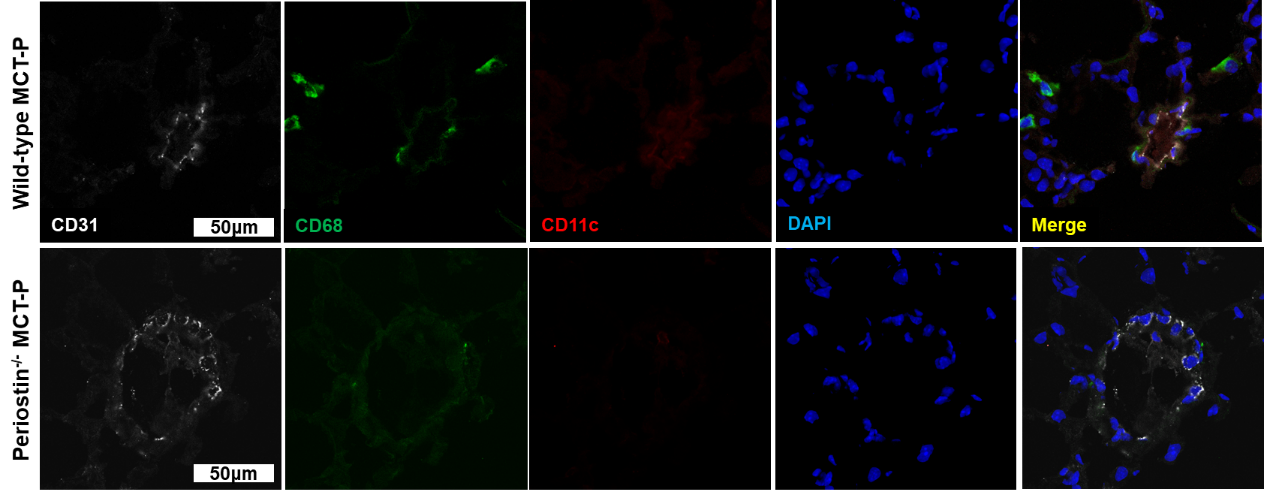


(A) Representative fluorescence immunostaining of CD31, CD68, the M1-marker CD11c, and DAPI in peri-pulmonary arteries from WT normoxia, WT SuHx, and periostin^-/-^ SuHx mice two weeks post-stimulation with Sugen5416 and hypoxic exposure (white, CD31; green, CD68; red, CD11c; blue, DAPI). Almost no CD11c-positive cells were observed in all groups.

(B) Representative fluorescence immunostaining of CD31, CD68, CD206, and DAPI in peri-pulmonary arteries from WT MCT-P, and periostin^-/-^ MCT-P mice (white, CD31; green, CD68; red, CD206; blue, DAPI). Quantitative analysis of the macrophage accumulation in the lungs of WT MCT-P and periostin^-/-^ MCT-P is shown. The percentage of CD68+ and CD206+ cells was calculated as a ratio to DAPI blue cells. Results are presented as means ± SD from 11–13 vessels per group. Statistical comparisons were conducted using Unpaired t test. *P<0.05.

(C) Representative fluorescence immunostaining of CD31, CD68, CD206, and DAPI in peri-pulmonary arteries from WT MCT-P, and periostin^-/-^ MCT-P mice (white, CD31; green, CD68; red, CD11c; blue, DAPI). Almost no CD11c-positive cells were observed in both groups.

**Figure S3. Cytokines induce periostin secretion in HPASMCs and HPMVECs**

**HPASMC**

**A**


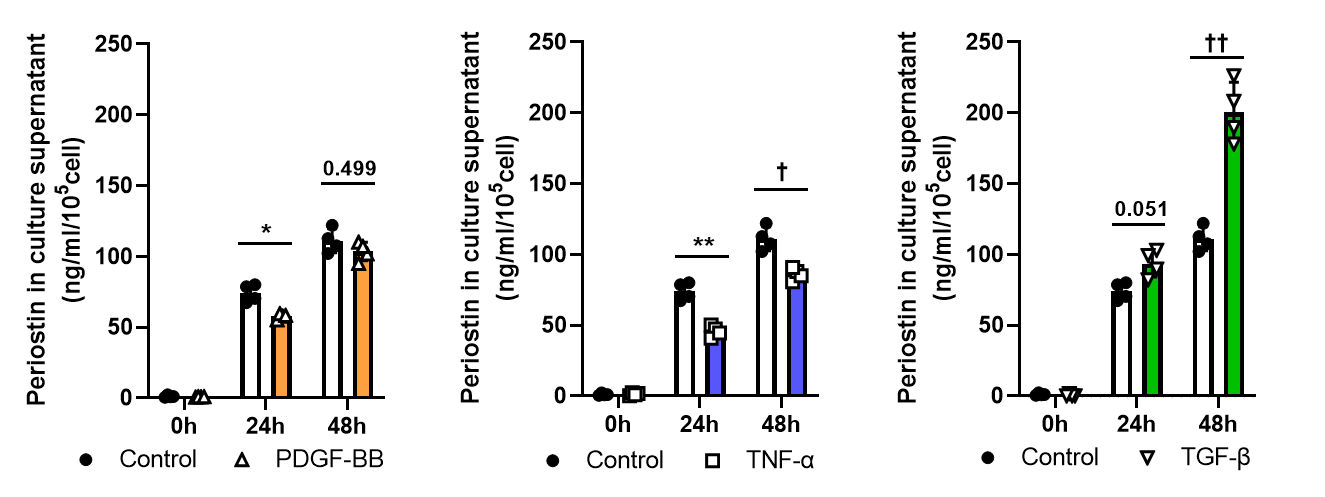


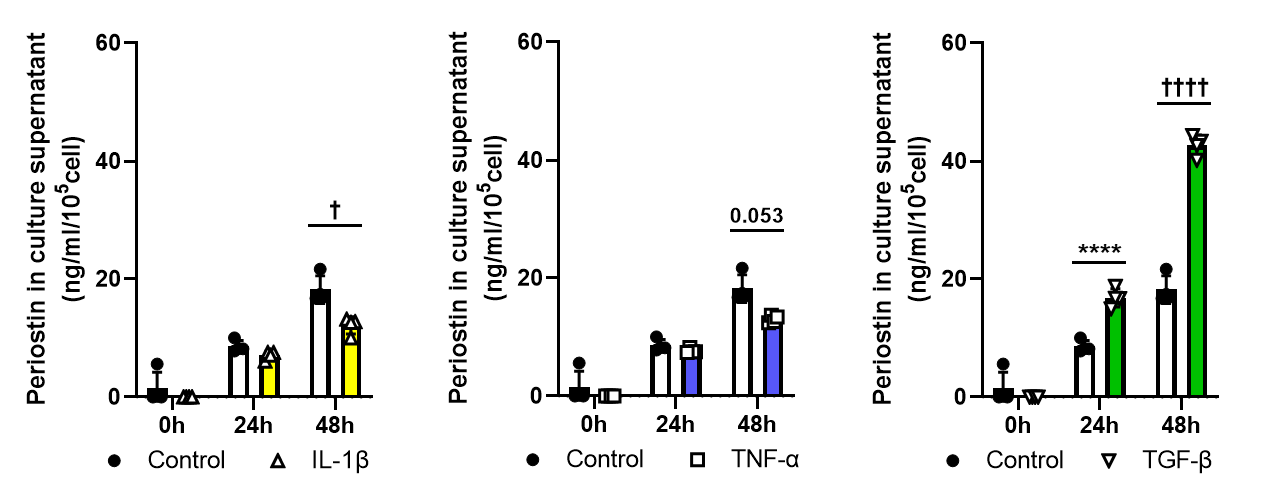


**HPMVEC**

**B**

(A, B) HPASMCs were treated with PBS (control), PDGF-BB (30 ng/ml), TNF-α (5 ng/ml), or TGF-β2 (2.5 ng/ml) for 0, 24, and 48 h. HPMVECs were treated with PBS (control), IL-1β (4 ng/ml), TNF-α (5 ng/ml), or TGF-β2 (2.5 ng/ml) for 0, 24, and 48 h. Periostin protein levels in culture supernatants were determined using ELISA; periostin concentration was normalized by cell number. Results are presented as mean ± SD from four samples. Statistical comparisons were conducted using 2-way ANOVA followed by Sidak's multiple comparisons test. *P<0.05, **P<0.01, ****P<0.0001; †P<0.05, ††P<0.01, ††††P<0.0001.

|  | **Healthy controls**  (n=10) | **PH**  (n=49) | **PH with periostin < 149.4ng/mL**  (n=21) | **PH with periostin > 149.4ng/mL**  (n=28) | ***P*** |
| --- | --- | --- | --- | --- | --- |
| Age (years) | 49.8±7.4 | 56.5±14.1 | 53.1±14.0 | 59.0±13.5 | .152 |
| Female, n (%) | 5 (50%) | 34 (69.4%) | 14 (66.7%) | 20 (71.4%) | .762 |
| Mean PAP, mmHg  PAWP, mmHg  CO, L/min  PVR, Wood units | NA  NA  NA  NA | 31.2±8.8  8.67±3.99  4.70±1.53  5.09±3.12 | 30.8±8.7  8.40±4.03  5.32±1.80  5.03±2.57 | 31.6±8.9  8.88±3.95  4.20±1.02  5.13±3.50 | .753  .697  .014  .919 |
| eRVSP, mmHg | NA | 45.0±17.9 | 42.0±16.7 | 47.3±18.5 | .317 |
| Periostin, ng/mL | 64.6±25.0 | 190.5±128.4 | 78.9±44.3 | 274.2±105.1 | <.0001 |
| Classification, n (%)  Group1  iPAH  CTD-PH  SSc  SLE  MCTD  others  Group2  Group3  COPD  IP  Group4  Group5 | NA  NA  NA  NA  NA  NA  NA  NA  NA  NA  NA  NA  NA | 32 (65.3%)  5 (10.2%)  28 (57.1%)  13 (26.5%)  5 (10.2%)  8 (16.3%)  2 (4.1%)  1 (2.0%)  8 (16.3%)  3 (6.1%)  5 (10.2%)  7 (14.3%)  1 (2.0%) | 13 (61.9%)  3 (14.3%)  10 (47.6%)  0 (0.0%)  4 (19.1%)  5 (23.8%)  1 (4.8%)  1 (4.8%)  5 (23.8%)  2 (9.5%)  3 (14.3%)  3 (14.3%)  0 (0.0%) | 20 (71.4%)  2 (7.1%)  18 (64.3%)  13 (46.4%)  1 (3.6%)  3 (10.7%)  1 (3.6%)  0 (0.0%)  3 (10.7%)  1 (3.6%)  2 (7.1%)  4 (14.3%)  1 (3.6%) | .547  .643  .263  .0002  .150  .263  >.999  .429  .442  .569  .643  >.999  >.999 |
| With IP | 0 (0%) | 17 (34.7%) | 5 (23.8%) | 12 (42.9%) | 0.229 |
| FVC, % pred  DLCO, % pred | NA  NA | 74.45±21.17  35.03±17.29 | 75.36±19.94  37.27±16.83 | 73.68±22.13  33.25±17.45 | 0.795  0.461 |
| Treatment, n (%)  　Prostacyclin  　PDE5i  　ERA  　sGC  　HOT  *Immunosuppressants | NA  NA  NA  NA  NA  NA | 15 (30.6%)  24 (49.0%)  23 (46.9%)  5 (10.2%)  18 (36.7%)  20 (40.8%) | 9 (42.9%)  12 (57.1%)  10 (47.6%)  2 (9.5%)  8 (38.1%)  10 (47.6%) | 6 (21.4%)  12 (42.9%)  13 (46.4%)  3 (10.7%)  10 (35.7%)  10 (35.7%) | .129  .393  >.999  >.999  >.999  .558 |

**Table S1. Baseline characteristics of patients with PH and healthy controls.**

Results are presented as the mean ± standard deviation. Statistical comparisons were conducted using Unpaired t test and Fisher's exact test.

PAP: pulmonary arterial pressure; eRVSP: estimated right ventricular systolic pressure; iPAH: idiopathic pulmonary hypertension; CTD-PH: connective tissue disease associated with pulmonary hypertension; SSc: systemic sclerosis; SLE: systemic lupus erythematosus; MCTD: mixed connective tissue disease; COPD: chronic obstructive pulmonary disease; IP: interstitial pneumonia; PDE5i: phosphodiesterase type 5 inhibitors; ERA: endothelin receptor antagonists; sGC: Soluble guanylate cyclase stimulators; HOT: Home Oxygen Therapy.

*Immunosuppressants include corticosteroids, azathioprine, cyclosporin, and tacrolimus.

**Table S2. Primers used for qPCR**

| Interleukin-6: forward  reverse | 5′-ACAAAGCCAGAGTCCTTCAGAGAGATAC-3′  5′-TGAATTGGATGGTCTTGGTCCTTAGCCA-3′ |
| --- | --- |
| Tumor necrosis factor-α: forward  reverse | 5′-GGTGCCTATGTCTCAGCCTCTT-3′  5′-GCCATAGAACTGATGAGAGGGAG-3′ |
| Interleukin-1β: forward  reverse | 5′-TGCCACCTTTTGACAGTGATG-3′  5′-CAAAGGTTTGGAAGCAGCCC-3′ |
| Fibroblast growth factor 2: forward  reverse | 5′-AAGCGGCTCTACTGCAAGAACG-3′  5′-CCTTGATAGACACAACTCCTCTC-3′ |
| C-C motif chemokine 2: forward  reverse | 5′-GCTACAAGAGGATCACCAGCAG-3′  5′-GTCTGGACCCATTCCTTCTTGG-3′ |
| C-C motif chemokine 4: forward  reverse | 5′-ACCCTCCCACTTCCTGCTGTTT-3′  5′-CTGTCTGCCTCTTTTGGTCAGG-3′ |
| C-C motif chemokine 7: forward  reverse | 5′-CAGAAGGATCACCAGTAGTCGG-3′  5′-ATAGCCTCCTCGACCCACTTCT-3′ |
| C-X-C motif chemokine ligand 1: forward  reverse | 5′-TCCAGAGCTTGAAGGTGTTGCC-3′  5′-AACCAAGGGAGCTTCAGGGTCA-3′ |
| C-X-C motif chemokine ligand 2: forward  reverse | 5′-CATCCAGAGCTTGAGTGTGACG-3′  5′-GGCTTCAGGGTCAAGGCAAACT-3′ |
| Found in Inflammatory Zone 1: forward  reverse | 5′-CAAGGAACTTCTTGCCAATCCAG-3′  5′-CCAAGATCCACAGGCAAAGCCA-3′ |
| Inducible nitric oxide synthase: forward  reverse | 5′-GAGACAGGGAAGTCTGAAGCAC-3′  5′-CCAGCAGTAGTTGCTCCTCTTC-3′ |
| 18S ribosomal RNA: forward  reverse | 5′-CGGAAAATAGCCTTCGCCATCAC-3′  5′-ATCACTCGCTCCACCTCATCCT-3′ |
