# Supplementary material for: Periostin‐related progression of different types of experimental pulmonary hypertension: A role for M2 macrophage and FGF‐2 signalling
Source: Respirology. 2022 Mar 22;27(7):529–38. doi: 10.1111/resp.14249 (PMC9313806; doi:10.1111/resp.14249)
Supplement: Supplementary file 2 — Visual Abstract. Periostin‐related progression of different types of experimental pulmonary hypertension: A role for M2 macrophage and FGF‐2 signalling [file RESP-27-529-s002.pdf]

Periostin-related progression of different types of experimental pulmonary hypertension: A role for M2 macrophage and FGF-2 signalling

Hypothesis:

Periostin, ECM and matricellular protein, contribute to the progression of experimental pulmonary hypertension (PH).

Sugen5416/Hypoxic (SuHx) PH

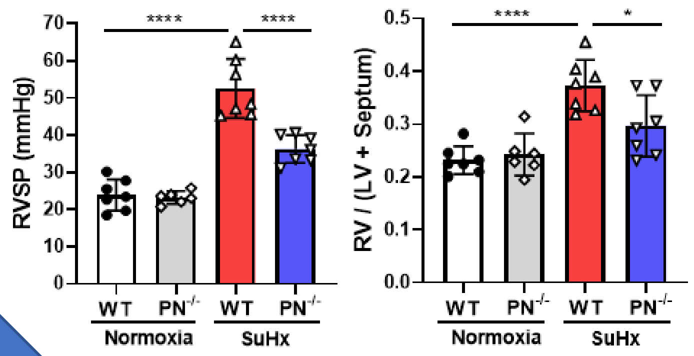

Monocrotaline-pyrrole (MCT-P) PH

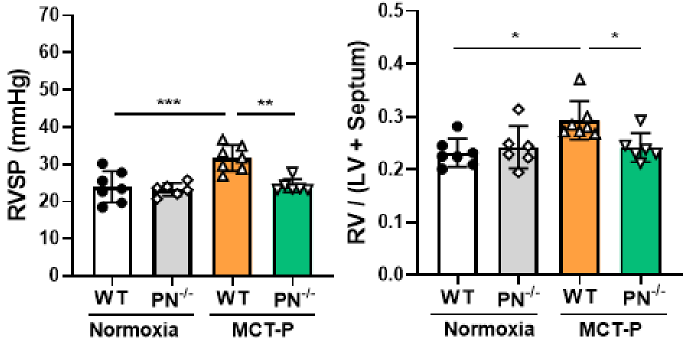

Wild type mice: WT, Periostin -/- mice: PN -/-

Peri-vascular accumulation of M2 macrophage in SuHx PH mice

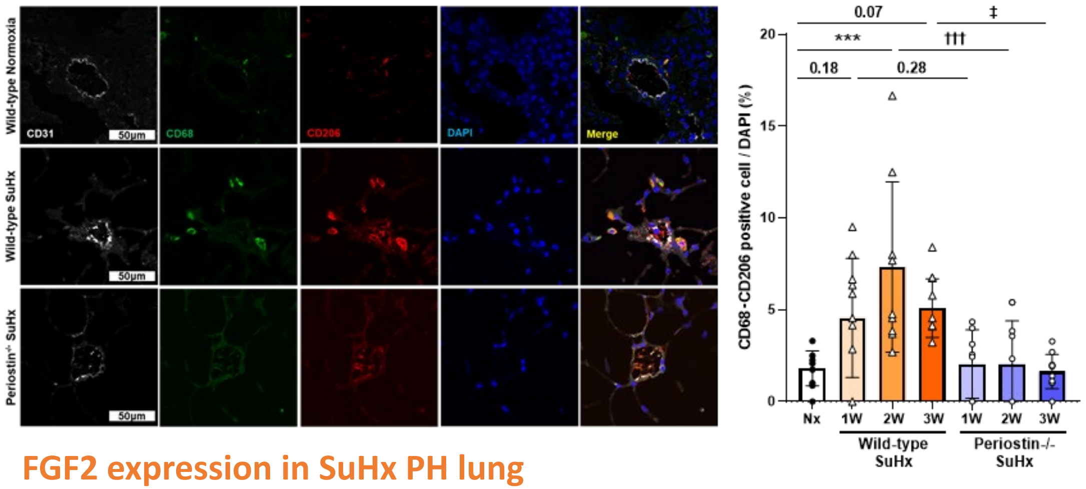

FGF2 expression in SuHx PH lung

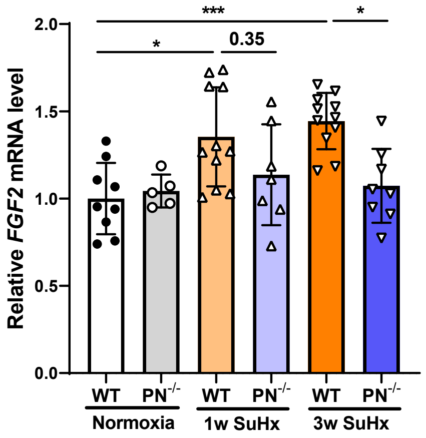

Conclusion:

Periostin-related early recruitment of M2 macrophage and subsequent enhancement of FGF-2 signalling may contribute to the progression of experimental PH.

Respirology

Periostin-related progression of different types of experimental pulmonary hypertension: A role for M2 macrophage and FGF-2 signalling

Yoshida et al. Respirology 2022; DOI:10.1111/resp.14249
